# Long-term written language experience affects grammaticality judgements and usage but not priming of spoken sentences

**DOI:** 10.1177/17470218211005228

**Published:** 2021-04-08

**Authors:** Saoradh Favier, Falk Huettig

**Affiliations:** 1 Max Planck Institute for Psycholinguistics, Nijmegen, The Netherlands; 2 Centre for Language Studies, Radboud University, Nijmegen, The Netherlands

**Keywords:** Literacy, grammaticality judgements, long-term syntactic priming

## Abstract

“Book language” offers a richer linguistic experience than typical conversational speech in terms of its syntactic properties. Here, we investigated the role of long-term syntactic experience on syntactic knowledge and processing. In a preregistered study with 161 adult native Dutch speakers with varying levels of literacy, we assessed the contribution of individual differences in written language experience to offline and online syntactic processes. Offline syntactic knowledge was assessed as accuracy in an auditory grammaticality judgement task in which we tested violations of four Dutch grammatical norms. Online syntactic processing was indexed by syntactic priming of the Dutch dative alternation, using a comprehension-to-production priming paradigm with auditory presentation. Controlling for the contribution of nonverbal intelligence quotient (IQ), verbal working memory, and processing speed, we observed a robust effect of literacy experience on the detection of grammatical norm violations in spoken sentences, suggesting that exposure to the syntactic complexity and diversity of written language has specific benefits for general (modality-independent) syntactic knowledge. We replicated previous results by finding robust comprehension-to-production structural priming, both with and without lexical overlap between prime and target. Although literacy experience affected the usage of syntactic alternates in our large sample, it did not modulate their priming. We conclude that amount of experience with written language increases explicit awareness of grammatical norm violations and changes the usage of (prepositional-object [PO] vs. double-object [DO]) dative spoken sentences but has no detectable effect on their implicit syntactic priming in proficient language users. These findings constrain theories about the effect of long-term experience on syntactic processing.

## Introduction

Syntactic diversity and complexity are key properties of “book language.” Sentence structure is often elaborate, with subordination, for instance, occurring 60% more frequently in written narratives than in spoken sentences (Kroll, 1977, cited by Kolinsky & Morais, 2018). Analyses of spoken and written corpora reveal pronounced asymmetry in the distributions of syntactic structures such as passives, object relative clauses, and participial phrases (e.g., Roland et al., 2007). It is important to note that exposure to the richer syntactic environment of “book language” can similarly be gained from listening to audiobooks or through shared reading for children. The associated benefits for syntactic knowledge can thus be considered a secondary influence of literacy, distinct from primary influences, which arise as a direct consequence of the physical act of reading (Huettig & Pickering, 2019, for further discussion). For example, Crain-Thoreson and Dale (1992) observed a secondary influence of “literate activity” in preliterate children. Their longitudinal study showed that the frequency of shared story reading with parents at 24 months reliably predicted performance on an auditory standardised test of syntactic comprehension at 30 months.

The current study investigated the contribution of individual differences in *lifelong* literacy experience to syntactic processes. Why is that an important question? We suggest that it is a crucial prediction of experience- and usage-based theories of cognitive processing that lifelong experience directly affects processing. In the domain of language, for example, it has been proposed that acquisition is shaped by the quality and quantity of the input a language user receives (e.g., Abbot-Smith & Tomasello, 2006; Bybee, 2006). “Book language” is a source of high-quality input, based on its increased syntactic complexity and diversity relative to conversational speech (Kroll, 1977; Roland et al., 2007). In terms of input quantity, skilled readers encounter a larger volume of language through reading more, in addition to processing information at a faster rate than is possible for listeners (e.g., skilled readers read English fiction at about 260 words per minute—approximately twice the typical speech rate; Brysbaert, 2019).

Indeed, there are a number of previous findings that suggest that lifelong literacy experience affects syntactic processes in spoken language, such as the use of syntactic cues in ambiguous pronoun interpretation (Langlois & Arnold, 2020) and prediction (Favier et al in press., Mishra et al., 2012). Dąbrowska (2012), moreover, reviewed experimental work investigating the syntactic abilities of adult L1 speakers with varying levels of education and reported converging evidence for considerable individual differences in knowledge of “core” grammatical constructions (including complementation, quantifiers, and passives, but see Favier & Huettig, in press). Differences were robustly correlated with education: Although high educational attainment groups tended to score at or near ceiling, performance among individuals with low educational attainment was often at chance. Regarding the underlying factor driving these effects, it was acknowledged that education could be acting as a proxy for print exposure. The two factors are of course intertwined (print exposure correlates with years of formal schooling, for example, Dąbrowska, 2018), and later work indeed revealed an independent contribution of print exposure to syntactic proficiency. Street and Dąbrowska (2010) found that print exposure reliably predicted comprehension of passives in a group of adults matched for educational attainment. Reading experience was a weaker predictor of performance on quantifier constructions in the same study, possibly reflecting the more symmetrical distribution of quantified noun phrases across spoken and written modalities (in contrast to full passives that occur 7 times more frequently in written texts). In later work, testing comprehension of a range of grammatical constructions frequently heard in everyday conversation, Dąbrowska (2018) observed a significant (albeit small) unique contribution of print exposure.

Comprehension is not the only domain in which lifelong written language experience can have consequences for syntactic processing. Montag and MacDonald (2015) examined the effect of prior reading experience on implicit sentence production choices in children and adults. Individuals who scored highly on the Author Recognition Test (ART; used as an index of print exposure) showed a pattern of production in their spoken language that reflected structural distributions in analysed written language corpora (specifically, increased frequency of passive relative clauses, which are rarely encountered in spoken language). This result leads straightforwardly to the conclusion that long-term exposure to a syntactic structure via reading facilitates its production in speech. The authors posited that becoming a reader entailed a quantitative and qualitative shift in linguistic experience, which continued to shape syntactic behaviour throughout adulthood. In short, there is considerable experimental evidence that lifelong literacy experience affects syntactic processes, both in spoken language comprehension and production.

Although experience- and usage-based theories of cognitive processing predict that lifelong experience directly affects syntactic processes, it is conceivable that not all syntactic knowledge and processing is affected by literacy experience to the same extent. We chose to explore the effect of long-term literacy experience on grammaticality judgements and syntactic priming because, to the best of our knowledge, there is little to no work that has investigated literacy-related influences on participant performance in these two experimental paradigms. Moreover, the two experimental tasks differ to the extent that they tap offline syntactic knowledge and online syntactic processing. It is important to point out that no psycholinguistic task involves purely explicit or purely implicit processes, but a mixture of both. It is, however, generally agreed that grammaticality judgement and syntactic priming are located at opposite poles of this continuum and it is conceivable that experience influences explicit processes differently than implicit ones.

Individual patterns of long-term syntactic experience related to literacy experience could conceivably play a role in individual differences in syntactic priming behaviour, for example, through shaping the base frequencies of structural alternates. Base frequencies have been shown to modulate structural priming in a number of previous studies (e.g., Bernolet & Hartsuiker, 2010; Jaeger & Snider, 2013; Segaert et al., 2016). In explaining the contribution of literacy to the grammaticality judgement task, some have argued that the decontextualised nature of written language facilitates metalinguistic thinking (e.g., Ravid & Tolchinsky, 2002, see Huettig & Mishra, 2014, for a review). Dąbrowska (2018) posited that inferring meaning from written text requires greater focus on the linguistic form because of the absence of extralinguistic cues typically available in speech (e.g., prosody and gesture). As well as being more conducive to learning syntactic structures, this attention to form may also support the “meta-syntactic” processes involved in grammaticality judgement. The idea that literacy brings with it an explicit analytical awareness of language itself is supported by evidence for the causal role of alphabetic literacy acquisition in meta-phonological abilities (e.g., phoneme deletion, Morais et al., 1979). For Kolinsky and Morais (2018), metalinguistic thinking is a key feature of the metaphorical “literate glasses” through which literate people perceive the world.

### Grammaticality judgement

Dutch is an interesting case study for grammaticality judgement because of the prevalence of syntactic forms that are prohibited by prescriptive grammar but nevertheless occur frequently in the daily speech of native Dutch speakers. Well-documented examples include the use of the object pronoun *hun* “them” as a subject, and the comparative marker *als* “as” in comparative constructions of inequality, where *dan* “than” is prescribed. Spoken corpus analyses reveal the prevalence of these prescriptive norm violations to be highest among low-educated speakers (Hubers & de Hoop, 2013; van Bergen et al., 2011).

### Syntactic priming

Syntactic (or structural) priming offers a tool to investigate online syntactic processing. Syntactic priming has more implicit components than metalinguistic tasks such as grammaticality judgement, though may involve some explicit components as well (e.g., Bernolet et al., 2016). Bock (1986) found that after hearing and repeating a sentence like “The corrupt inspector offered a deal to the bar owner,” participants were more likely to use a prepositional-object (PO) dative to describe an unrelated pictured event (e.g., “The boy is handing a valentine to the girl”), compared with its alternative, the double-object (DO) dative (“The boy is handing the girl a valentine”). Since it was first reported over 30 years ago, the effect of recent syntactic experience on subsequent production has been demonstrated with a variety of tasks, syntactic structures, and languages (see Mahowald et al., 2016, for a meta-analysis). Evidence from preliterate children (e.g., Branigan & McLean, 2016) shows that syntactic priming can occur without reading experience. However, it is particularly interesting for the purpose of the present study because it has been described both as a short-term (e.g., Pickering & Branigan, 1998) and a long-term phenomenon (e.g., Chang, 2002; Chang et al., 2000, 2006).

There are many different theoretical accounts of syntactic priming, but a main distinction can be made between activation-based accounts and error-based learning accounts. Traditional activation-based accounts are more compatible with short-term activation. Pickering and Branigan’s (1998) account of syntactic priming, for example, posits that verb lemmas and their associated combinatorial nodes (specifying structure) become activated during comprehension, and that residual activation in a given combinatorial node increases the likelihood of reproducing a recently encountered structure (Pickering & Branigan, 1998). Due to the rapid decay of residual activation, syntactic priming according to this account is a relatively short-term phenomenon. Error-based learning accounts, in contrast, assume that syntactic priming is a more long-term phenomenon. Chang and colleagues (Chang, 2002; Chang et al., 2000, 2006), for example, propose that during comprehension, the system continuously updates the weighting of mappings between message-level and abstract syntactic representations according to the input it receives. This implicit learning model of syntactic priming thus predicts long-term effects of experience on syntactic priming. In line with such an account, it has been observed that syntactic priming can persist over multiple intervening sentences (Bock et al., 2007; Bock & Griffin, 2000) and even a week (Branigan & Messenger, 2016).

An interesting and important finding is that syntactic priming effects are much larger when the verb is repeated across prime and target (the so-called lexical boost effect, for example, Pickering & Branigan, 1998; Traxler et al., 2014). We believe it is fair to say that researchers (especially those in the error-based learning camp) have struggled to reconcile the lexical boost effect (typically assumed to be a lexical short-term memory phenomenon) with (nonverb repeated) syntactic priming (typically assumed to be based on general structural procedures, that is, rules). To reconcile long-term persistence with the short-lived boost to syntactic priming that occurs when prime and target sentences share a lexical head (e.g., Hartsuiker et al., 2008), for example, it has been proposed that repeated lexical material may simply cue retrieval of the prime sentence (Bernolet et al., 2016).

A (arguably) more “natural” account of the lexical boost effect in syntactic priming is to abandon the traditional distinction between words and rules. Jackendoff and colleagues (Huettig et al., under review; Jackendoff, 2002; Jackendoff & Audring, 2020) argue that all rules can be restated in schema form and, as a consequence, take on the same format as words, with the only difference to words being that some of a schema’s structure is made up of variables (see Jackendoff & Audring, 2020, for a detailed linguistic discussion of this). In this approach, words and schemas belong to a (single system) extended lexicon (see E. Bates & Goodman, 1997; Fillmore, 1988; Langacker, 1987, for similar views). Words and syntactic schemas can prime subsequent occurrences, and accordingly, the lexical boost effect is a natural consequence of activation spreading between words and schemas as both are pieces of stored linguistic structure that are boosted by recent usage. This account also provides a natural explanation of the finding that low-frequency items prime more than high-frequency items (the so-called inverse priming effect, for example, Scheepers, 2003; Snider & Jaeger, 2009) because a frequently encountered item has a high resting state activation, which requires more input activation to raise its resting state activation (by the same amount) than a low-frequency item (see Huettig et al., under review, for further discussion).

It is important to point out here that long-term persistence of syntactic priming in the literature refers to priming over multiple intervening sentences or at maximum a week. To our knowledge, it has not been directly explored whether *lifelong* experience with alternating structures has an influence on syntactic priming. Lifelong written language experience, for instance, may influence the usage of alternating structures if a given alternate is more prevalent in print materials. In the present study, we measure the potential bias associated with literacy experience directly by using a baseline measure of Dutch dative usage (PO or DO) in people with varying literacy levels (rather than relying on the small corpora that are available for Dutch, which are prone to biases). If literacy experience changes the usage of Dutch dative alternates, then it is conceivable that this affects their priming. For instance, in the account of language processing proposed by Jackendoff and colleagues, infrequent structures get more of a boost than frequent structures from the same amount of activation, resulting in stronger priming. This is because more activation is required to raise the resting activation of a frequent structure (perhaps reaching ceiling asymptotically). In short, reduced written language experience may make certain structures more infrequent for individuals with lower literacy and thus potentially result in greater priming of those structures.

### The current study

The current study investigated the contribution of individual differences in literacy experience to offline and online syntactic processes, as indexed by grammaticality judgement and syntactic priming, respectively. Although the majority of participants in psycholinguistic research to date have been university students, this group is unrepresentative of the general population in terms of language and literacy skills, which are likely to be skewed towards the upper end of the normal distribution. Given the theoretical importance of sampling from a broad spectrum of literacy abilities (Tarone & Bigelow, 2005), we focused our efforts on recruiting participants from diverse educational backgrounds. In addition, we tested participants outside of the lab to facilitate community participation.

We integrated correlational and experimental methods, using a correlational design with literacy as a predictor and grammaticality judgement accuracy and syntactic priming magnitude as the predicted variables. We measured a range of literacy-related skills as predictors: word and pseudo-word reading, receptive vocabulary knowledge, misspelling detection, author name recognition, and self-reported reading habits. We performed principal components analysis on these six variables to derive a principal component score, providing an index of literacy for our correlational analyses. We also included tests of working memory capacity, processing speed, and nonverbal intelligence in our battery. These served as covariates in the analyses.

We developed an auditory grammaticality judgement task to probe participants’ knowledge of four prescriptive grammatical norms in Dutch, specifically their sensitivity to norm violations that occur in the everyday speech of many native speakers (Hubers et al., 2016). Whereas previous studies have investigated the grammaticality of these predominantly spoken constructions via the written modality, we used auditory presentation as we aimed to assess the effect of written language experience on spoken language processing. The task required participants to make a binary normative judgement about the syntactic form of each utterance (correct/incorrect). The within-subjects manipulated variable was grammaticality: whether or not the stimulus sentence violated a Dutch grammatical norm. Our outcome measure was the proportion of experimental items correctly judged as grammatical or ungrammatical, according to prescriptive usage. We predicted that native speakers’ grammaticality judgements are influenced by their awareness of the syntactic discrepancies between written and spoken Dutch. We assumed that this awareness correlates with reading experience (i.e., exposure to written language) as indexed by our literacy measures. Put another way, prescriptive grammatical norms are reliably attested in written language, whereas everyday spoken Dutch frequently contains violations of prescribed usage. Therefore, on the basis of differing input, we predicted that participants with less reading experience would have more difficulty recognising prescriptive norm violations (i.e., their judgements would be more likely to reflect the syntactic patterns of spoken language). A secondary prediction was that grammaticality judgement would correlate positively with vocabulary knowledge, in line with the close association observed between grammar and vocabulary in development (E. Bates et al., 1995; Hayiou-Thomas et al., 2006).

The syntactic priming experiment focused on the Dutch dative alternation, using a comprehension-to-production paradigm (following Bernolet & Hartsuiker, 2010). Participants alternated between listening to (prime) sentences, performing a picture verification task, and providing spoken responses to target pictures. Rather than generating dative sentences from written verbs, participants in the current study completed dative sentence stems that were presented auditorily. This more constrained elicitation format was intended to minimise the involvement of literacy-related abilities in the task. Primes and their corresponding target pictures were adjacent, as immediate priming effects are expected to be stronger than priming after a lag (Bernolet et al., 2016). We manipulated the structure of the prime (PO or DO dative, within items) and the repetition of the head verb between prime and target (verb same or different; within subjects). In line with previous research, we predicted that primed structures would be produced more frequently in the priming conditions. Furthermore, we predicted an increased likelihood of producing the primed structure when the prime verb was repeated in the target sentence (lexical boost). By including a baseline measure (rather than relying on the limited availability of Dutch corpora, Colleman, 2009; Haemers, 2012), we directly measured whether written language experience affects usage (base levels) of Dutch PO/DO dative constructions, and in turn their priming. Finally, based on the account presented by Jackendoff and colleagues, we predicted that reduced written language experience makes some structures more infrequent for individuals with lower literacy and thus results in stronger priming of those structures. For the same reason, we predicted that reduced written language experience also results in an increased lexical boost (under the assumption that low literates will get comparatively less exposure to certain verbs than high literates).

### Preregistered predictions

Literacy will be positively correlated with accuracy in an auditory grammaticality judgement task (directional).

Vocabulary knowledge will correlate positively with grammaticality judgement accuracy (directional).

Participants will produce more target completions containing the primed structure after hearing a prime sentence versus a structurally unrelated control sentence (directional).

The likelihood of producing the primed structure will be enhanced when the prime verb is repeated in the target sentence (lexical boost) (directional).

We predict a negative correlation between literacy and the magnitude of the syntactic priming effect observed (directional).

The lexical boost will be stronger in participants with lower literacy (directional). (see https://osf.io/zykp2)

## Method

The study was preregistered with the Open Science Framework, including a sample size appropriate to its correlational, individual differences design. The sampling rationale was based on work by Schönbrodt and Perugini (2013), in which Monte Carlo simulations of correlational analyses identified *N* = 161 as a point of stability for estimated correlation magnitudes, after which sample estimates do not deviate from a predefined “corridor of stability” around the true population value.

### Participants

A total of 161 Dutch native speakers participated for €10 per hour. We recruited a community-based sample through online and local advertising in Nijmegen, the Netherlands. A total of 20 participants were recruited and tested in their local public library. Email invitations were also sent to eligible 18- to 35-year-old native Dutch speakers in the Max Planck Institute’s participant database. None of the participants had a diagnosed reading disability and all had normal or corrected-to-normal hearing and vision. A total of 13 participants were excluded from the analysis: 11 scored less than 2.5 standard deviations below the sample mean on at least one of the individual difference measures, and two had missing data.

## Materials

### Individual difference measures

#### Literacy-related abilities

We developed a battery to assess a range of literacy-related abilities, both directly and indirectly. The battery comprised standardised assessments that have been widely used in the psycholinguistics literature, and some measures developed for the current study. Each is briefly described below.

##### Een Minuut Test

We administered a standardised test of word reading ability, consisting of 116 Dutch words that progressively increase in difficulty (Brus & Voeten, 1973). We instructed participants to read the list aloud from top to bottom, as quickly as possible. The score was the number of words read accurately in 1 min, precisely as printed on the test sheet. The experimenter timed the test using a stopwatch and scored responses online.

##### Klepel Test

We used a standardised test of pseudo-word reading ability, comprising 116 Dutch pseudo-words of progressively increasing complexity (van den Bos et al., 1994). The administration and scoring procedure were as above, except that participants had 2 min to read aloud as many items as accurately as possible. As some participants completed the list in less than 2 min, we also kept a record of their score after 1 min. Digital voice recordings of both reading tests were made, and a native speaker later verified the scores.

##### Peabody Picture Vocabulary Test (PPVT)

A large body of research highlights the bidirectional relationship between vocabulary knowledge and reading (e.g., Braze et al., 2007; Lee, 2011; Tannenbaum et al., 2006). In adulthood, most new words are encountered in written texts (Cunningham & Stanovich, 1998; Stanovich et al., 1995), making receptive vocabulary knowledge a useful proxy for literacy experience (not only oral language competence). We used a computerised version of the Dutch PPVT (Dunn et al., 2005). Each trial comprised a spoken word and a visual display with four numbered line drawings. Participants selected the picture that illustrated the word’s meaning by pressing the corresponding number on the keyboard. The task was self-paced and participants could listen to each word more than once. Trials were presented blocks of 12, which progressively increased in difficulty. If the number of incorrect responses in a block exceeded seven, the test was discontinued. The raw score was the final item number reached, minus the total number of errors. From this the participant’s standardised score and percentile rank were derived, based on Dutch age norms.

##### Misspelling Detection Test

We developed a short paper test to assess receptive spelling knowledge, based on norms from Dutch and Flemish university students (Marc Brysbaert, personal correspondence). We selected a subset of 20 high-prevalence words with item scores that correlated the most with total test scores (.30–.55 correlation). We chose words from the higher end of the item score distribution (0.87–0.99 correct), to account for the wider range of ability in our community-based sample relative to the norming sample. Each correctly spelled word had a misspelled counterpart featuring a single substitution error, for example, *onbemindt (correct spelling: onbemind). Two counterbalanced, pseudo-randomised lists were constructed such that all 20 words appeared in their correct and incorrect versions across the two lists and no more than three of the same condition appeared consecutively. Each word was presented in a plausible sentence context, for example, Hij stierf onbemind. We instructed participants to indicate whether the underlined word in the sentence was correctly spelled or not by marking a tick or a cross on the test sheet.

##### ART

The ART (Stanovich & West, 1989) is widely used as a proxy for engagement in print-related activities. Adapted for the Netherlands and Belgium (Brysbaert et al., 2013), the test comprises 60 author names, known to 66% of the Dutch norming sample, and 30 nonauthor foils that yielded 13% false alarms. We instructed participants to indicate which authors they knew and advised them against guessing as false alarms would be penalised. The test was completed on paper and untimed. The score was the number of authors correctly identified, minus the number of foils marked.

##### Reading Habits Questionnaire

A paper questionnaire was used to evaluate self-reported engagement in print-related activities. This was a Dutch translation of the subtest “Your Reading Activities,” extracted from the Organisation for Economic Co-operation and Development (OECD, 2009) Programme for International Student Assessment (PISA). Importantly, the questionnaire also probed time spent reading digital and online media. Participants answered questions on a 4- or 5-point Likert-type scale and the score was the sum of coded responses.

#### Covariates

We also administered a battery of covariate measures to assess nonverbal intelligence, processing speed, and verbal working memory.

##### Raven’s progressive matrices

To assess participants’ nonverbal intelligence, we administered a shortened, computerised version of Raven’s advanced progressive matrices test (RPM Raven et al., 1985). The task was to indicate via mouse-click which of eight shapes completed a matrix of geometric patterns. Participants had 20 min to complete 36 items. It was possible to skip any item and return to it at the end of the test. The score was the total number of correct responses.

##### Letter comparison

As an index of processing speed, we used the letter comparison task (based on Salthouse & Babcock, 1991). Participants were presented with pairs of capital letter strings containing only consonants, in large black font on a white screen. The task was to indicate whether the strings were the same or different by pressing “1” or “0,” respectively, on the keyboard. Half of the items consisted of three-letter strings and the other half six-letter strings. Incongruent pairs differed by only one letter. There were six practice trials and 48 test trials, each beginning with a fixation cross, followed by a pair of letter strings that remained on the screen until the participant responded. There was an intertrial interval of 1,000 ms. The score was calculated as the mean response time (RT) for all correct responses that were no more than three standard deviations slower than the participant’s grand mean RT.

##### Backward digit span

We used a computerised version of the backwards-recall digit span task to measure working memory capacity, with auditory presentation of stimuli (adapted from the Wechsler Adult Intelligence Scale; Wechsler, 1997). Participants listened via headphones to sequences of two to eight digits, spoken by a female Dutch native speaker with a consistent rate (1-s pauses) and neutral prosody. The task was to type the sequence heard in reverse order, using the keyboard. There were 14 test trials, comprising seven blocks of two trials. Between blocks, sequences increased in length by an increment of one digit. The test was discontinued if participants responded incorrectly to both items in a block. The score was the number of correctly recalled digit sequences. We also recorded a top recall score for each participant; that is, the number of digits in the longest correctly recalled sequence.

### Grammaticality judgement

We based our stimuli on previous work by Hubers et al. (2016), which focused on the perception of prescriptive grammatical norm violations in Dutch. For that study, they pretested several hundred sentences containing violations of five prescriptive norms. We thus had access to grammaticality ratings from an educationally diverse sample (*n* = 97; aged 18–35). We excluded one type of violation that was only relevant to written language and calculated difficulty scores for items in the remaining four categories: als/dan; mij/ik; hun/ze; die/dat. As the goal was to develop a task challenging enough to yield a spread of scores, we selected the eight lowest-scoring items from each category, after outliers were excluded. Accuracy scores in the final shortlist ranged from 0.50 to 0.94, with “die” constructions scoring the highest, and “hun” constructions the lowest.

For each category of norm violation, we devised eight control sentences featuring prescribed usage of the relevant critical word; als, mij, hun, or die (see Table 1). The resulting 64 critical sentences were matched for syllable length, critical word position, and the frequency and prevalence of lexical items.

**Table 1.** Example items from each category of norm violation with matched control sentences.

Critical word	Norm violation	Control
*hun*	Vorige week liepen **hun** naar de speeltuin. [Last week them walked to the playground]	Gisteren heb ik **hun** twee boeken gegeven. [Yesterday I gave them two books]
*als*	De jongen eet minder **als** zijn grote neef. [The boy eats less as his big cousin]	Zij is net zo groot **als** Vera op die hoge hakken. [She is just as tall as Vera in those high heels]
*mij*	Steven heeft eerder dan **mij** zijn rijbewijs gehaald. [Steven got his drivers licence earlier than me]	Hij vindt Linda aardiger dan **mij** maar niet grappiger. [He finds Linda kinder than me but not funnier]
*die*	Is er een bureau **die** voor mij bedoeld is? [Is there a desk that^a^ is meant for me?]	Kent Kees een supermarkt **die** nog goedkoper is? [Does Kees know a supermarket that is even cheaper?]

a Norm violation does not translate due to lack of grammatical gender in English.

Given the evident uncertainty among many native speakers of Dutch regarding the prescribed usage of these forms, we expected to see inaccuracy, both in the rejection of sentences that adhered to grammatical norms and in the acceptance of sentences that violated them.

In addition, we generated 16 filler sentences, half of which were “truly ungrammatical,” featuring syntactic anomalies consistently detected by Dutch native speakers, for example, errors relating to subordinate clause word order, or verb tense and number agreement. These unambiguous filler sentences allowed us to ensure that participants were not responding randomly.

Stimuli were recorded by a female native speaker of Dutch, using a Sennheiser ME64 microphone. The speaker was instructed to maintain natural, conversational speech rate and prosody across all items.

All 80 items were presented to all participants in one, pseudo-randomised list, such that no more than three correct or incorrect items appeared consecutively. A maximum of two consecutive sentences could contain the same critical word, but they always contrasted in terms of grammaticality. The task began with two filler trials, one ungrammatical. A full stimulus list is provided in the online supplementary materials.^1^

### Syntactic priming

We selected 10 alternating dative verbs that have previously yielded syntactic priming effects in Dutch (Bernolet et al., 2016; Bernolet & Hartsuiker, 2010; Hartsuiker et al., 2008). We used these target verbs to generate 10 sentence stems (e.g., *Hij geeft* [*He gives*], *Ze overhandigt* [*She hands over*]), which could be completed as either a PO or DO dative construction. The gender of the subject pronoun was balanced across items, with 50% of sentence stems using *ze* [she].

From the MultiPic database (Duñabeitia et al., 2018), we selected colour pictures of 10 inanimate and 10 animate nouns, matched for Log10 word frequency (SUBTLEX-NL; Keuleers et al., 2010), syllable length, picture naming agreement, and visual complexity. We used the two sets of pictures to generate 30 different theme-recipient pairs and assigned each pair to one of the 10 target sentence stems. We conducted a Google Books search to ensure that the transitional probabilities of target verb and animate/inanimate noun combinations were matched within items.

**Figure 1.**
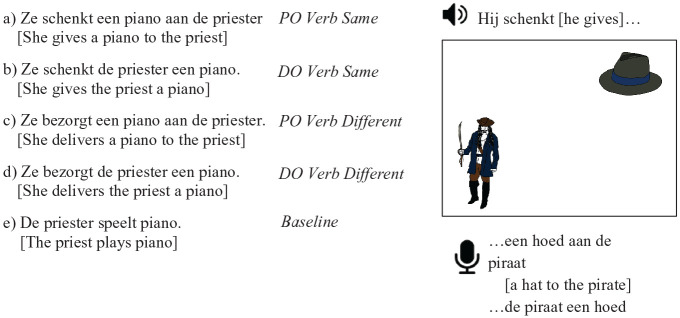
Example prime sentences with corresponding target picture, sentence stem, and expected completions. *Schenken* means “to give” (as in a gift).

For each target item, we constructed five prime sentences, corresponding to the following prime conditions: (a) PO Verb Same, (b) DO Verb Same, (c) PO Verb Different, (d) DO Verb Different, and (e) Baseline (see Figure 1).

We selected a separate set of 10 inanimate and 10 animate nouns from the MultiPic database, which provided 30 different combinations of prime themes and recipients. Prime sentences in Conditions (a) and (b) repeated the 10 target verbs. For priming Conditions (c) and (d), we selected 10 additional alternating dative verbs, on the basis of corpus and experimental data (Colleman, 2009; Colleman & Bernolet, 2012, more details provided in the online supplementary materials, see Note 1). For the Baseline Condition (e), we combined monotransitive and intransitive verbs with the same set of nouns, to generate sentences that were syntactically unrelated to the dative. Like the target stems, all dative prime sentences featured *hij* or *ze* as the agent of the dative action. The gender of the pronoun alternated between prime sentences and their corresponding target stems.

Auditory stimuli were recorded by a female Dutch native speaker, using a Sennheiser ME64 microphone. To create the set of target stems, we recorded both PO and DO versions of the complete dative sentences and cut them down using Praat software (Boersma & Weenink, 2017). This resulted in two versions of the same sentence stem for each of the 10 target verbs. We counterbalanced the PO and DO versions across target items, to reduce any differential influence of prosodic cues on participants’ syntactic choices.

We constructed 90 filler items that were syntactically and semantically unrelated to experimental items. To reduce the salience of the dative alternation, filler sentences varied in structure. Of the 90 filler items, 70 were simple transitives and intransitives in the present tense and the remaining 20 were complex sentences with a complement clause in the past tense, for example, *Hij zei dat het hemd werd gestreken* [*He said that the shirt was ironed*]. As auxiliary-participle word order is reversible in Dutch subordinate clauses, we constructed a set of complex sentences using both word orders, counterbalanced across experimental lists. Filler trials followed the same two-part structure as experimental trials: (a) sentence comprehension (picture verification) and (b) sentence completion (picture description). For the purposes of the cover task, 60% of items in the verification set featured a semantic mismatch between the sentence stimulus and the content of the visual display. For example, in one incongruent filler trial, participants saw a display with a man and a glass of milk and heard the sentence *De man drinkt koffie* [*The man drinks coffee*]. All dative trials were congruent.

The picture description set comprised noncritical pictures and corresponding sentence stems that would be unlikely to elicit a dative construction, for example, *De vrouw draagt* [*The woman wears*]. The precise format of elicitation varied, depending on the type of filler. For example, where participants were required to complete a sentence with a verb phrase or noun phrase, they heard part of the target phrase immediately before the sentence stem was presented, for example, *Rode. Hij slaapt in een* . . . [*Red. He sleeps in a* . . .], or *Geslapen. Ze zei dat de koala* . . . [*Slept. She said that the koala* . . .]. Participants were familiarised with the different types of fillers through practice trials at the start of the priming experiment.

We constructed five pseudo-randomised lists of critical stimuli, such that across the lists every item appeared once in each of the five experimental conditions. To give a more reliable measure of participants’ structural biases when not primed, we included six additional items in the neutral baseline condition, bringing the total number of dative trials to 36. These were interleaved with the 90 filler items, creating five lists of 126 trials, which we presented in a pseudo-random order such that each dative trial was preceded by at least two filler trials.

#### Procedure

Participants individually attended two sessions within the same week, each lasting approximately 1 hr. The first session consisted of the syntactic priming experiment, followed by the grammaticality judgement task. In the second session, participants completed the following sequence of tasks in the same order: Een Minuut Test, Klepel Test, Backward Digit Span, Letter Comparison, PPVT, Misspelling Detection Test, RPM, ART, and Reading Habits Questionnaire. Computerised tasks were carried out on a PC in a soundproofed experiment booth at the Max Planck Institute or on a laptop in a reserved quiet room in the public library. Participants completed the remaining tasks at a desk, under the supervision of the experimenter. Alternating between the two types of activities was intended to help sustain attention levels and balance task demands.

The grammaticality judgement task was carried out on a PC or laptop, with auditory stimuli presented via headphones. Participants were instructed to listen to each sentence and respond to the question, “Is dit een correcte Nederlandse zin?” (Is this a correct Dutch sentence?), by pressing “1” or “0” on the keyboard (for yes and no, respectively), and to guess if they were unsure. Each sentence was presented once, along with a visual prompt showing “ja = 1” on the left of the screen and “nee = 0” on the right. There was no time limit on responses and no feedback given. As soon as a button press was recorded, the screen “Volgende zin” appeared and the next trial began. The task took 10–15 min to complete.

The syntactic priming experiment began with written instructions, followed by a series of examples to illustrate the verification task and demonstrate how sentence stems were to be completed in the picture description part of the trial. These demonstrations featured prerecorded responses from a Dutch native speaker. Three of the example dative trials used PO target completions, and three DO, so that participants’ exposure to the two structures was balanced before beginning the priming experiment. The dative example trials were interleaved with transitive filler examples to reduce the salience of the dative alternation. Pilot testing indicated that such a demonstration was necessary to ensure that experimental stimuli reliably elicited dative responses. The passive demonstration phase was followed by four active practice trials, which were semantically and syntactically unrelated to the subsequent experimental trials. At the start of each trial, participants saw a fixation cross, followed by a pair of pictures, positioned in the lower left and upper right corner of the visual display (see Figure 1). The position of the animate and inanimate pictures on the screen was counterbalanced across all items and randomised within each experimental list. Participants then heard a prerecorded prime sentence that referred to the displayed pictures. As a cover task, they were instructed to press “1” or “0” on the keyboard to indicate whether the content of the sentence and the picture were, respectively, congruent or incongruent. Participants received immediate on-screen feedback: “Correct!” or “Helaas, volgende keer beter!” (Better luck next time!). In the case of a correct response, the feedback screen also displayed reaction time in milliseconds (intended to increase motivation and engagement with the task). The second part of the trial comprised a new visual display with two target pictures (semantically unrelated to the prime pictures) and an auditorily presented dative sentence stem. Participants completed the sentence aloud with either a PO or DO construction by naming the theme and recipient displayed. To reduce any influence of looking bias on their syntactic choices, participants had a 1,000 ms preview of the visual display before they heard the target sentence stem. Responses were recorded via a microphone attached to the headset.

## Results^2^

### Scoring

Correct responses in the grammaticality judgement task were coded as “1” and incorrect responses as “0.”

Responses in the syntactic priming experiment were manually coded as PO datives, DO datives, or Others. A response was coded as PO if the theme of the action was supplied first, followed by the preposition *aan* (to) and the recipient (e.g., after the target stem *Hij schenkt* [*He gives*] in Figure 1, *een hoed aan de piraat* [*a hat to the pirate*]). A response was coded as DO if the recipient was supplied first with no preposition, followed by the theme of the action (e.g., after the same target stem, *de piraat een hoed* [*the pirate a hat*]). Nondative responses were coded as Other.

### Descriptive summary of individual difference measures

Means, standard deviations, and ranges for each measure are reported in Table 2, as well as a descriptive summary of age. Correlations among the individual difference measures can be found in the online supplementary materials (Table S2, see Note 1).

**Table 2.** Means, standard deviations, ranges, and maximum possible scores for the individual difference measures (*N* = 148).

Measure	*M*	*SD*	Range	Max
Literacy related				
1. Word reading (Een Minuut)	94.68	14.60	56−116	116
2. Pseudo-word reading (Klepel)	104.90	9.83	77−116	116
Klepel 1 min	65.69	10.76	40−94	–
3. Vocabulary (PPVT)	101.20	10.56	74−128	139
PPVT percentile rank	53.18	23.73	4−97	100
4. Misspelling detection	18.66	1.37	14−20	20
5. Author recognition	8.12	7.74	0−50	60
6. Reading habits questionnaire	80.04	8.88	41−105	114
Covariates				
7. Nonverbal IQ (RPM)	19.72	5.91	5−32	36
8. Processing speed (LC)	1,076.00	191.51	673−1,644	–
9. Working memory (BDS)	7.90	2.15	2−13	14
BDS top recall	5.45	1.23	2−8	8
10. Age (years)	23.41	3.44	18.00−34.58	–

Max: Maximum possible score; PPVT: Peabody Picture Vocabulary Test; RPM: Raven’s Progressive Matrices; LC: Letter Comparison task; BDS: Backward Digit Span task.

### Principal components analysis

**Figure 2.**
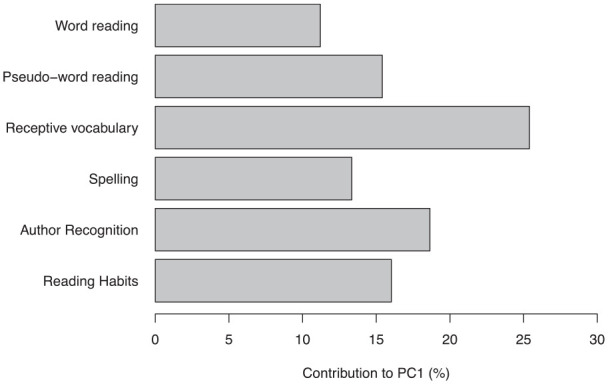
Bar plot showing the contribution of individual variables to the first principal component for literacy (PC1).

Our test battery targeted a range of skills involved in literacy (Measures 1−6 in Table 2). Using the *FactoMineR* package in R (Lê et al., 2008; R Core Team, 2012), we performed principal components analysis on this subset of variables to derive an underlying construct that explained the maximal amount of variance in the literacy data. The *FactoMineR* package contains a built-in function to evaluate the intercorrelation of variables with respect to a predefined criterion. The analysis extracted six principal components, of which the first explained 37.7% of the variance in the data. The composition of the first principal component is shown in Figure 2. All six literacy-related measures make some contribution, most of all receptive vocabulary (Measure 3, Table 2) at 25%. We use the first principal component score as an index of literacy experience (predictor variable) in the main analyses as it explains the largest portion of variance in literacy-related skills. Further details about the other five principal components can be found in the online supplementary materials (see Note 1).

### Grammaticality judgement

**Figure 3.**
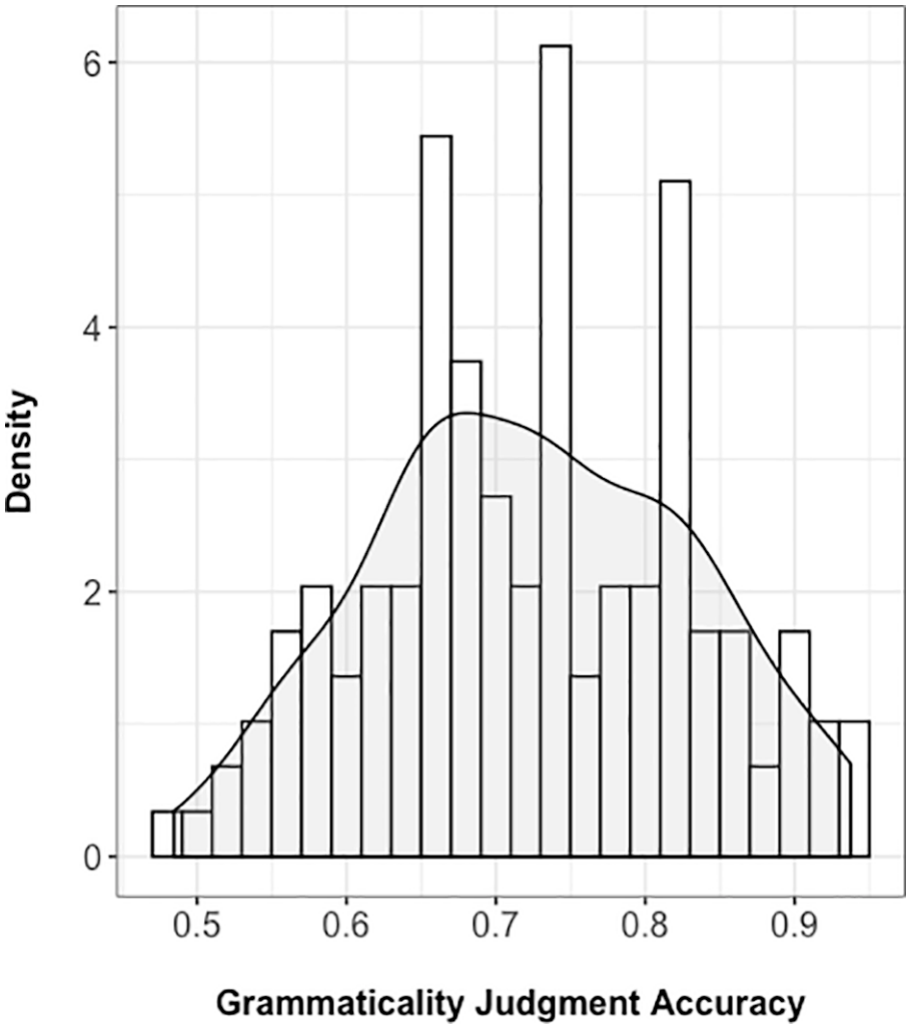
Histogram of grammaticality judgement accuracy with a plotted density curve.

Descriptive statistics on the grammaticality judgement task were 0.72 (mean), 0.11 (standard deviation), 0.72 (median), and 0.48−0.94 (range). Consistent with the equal mean and median values, the histogram in Figure 3 reflects a fairly symmetrical distribution of scores across the sample. Only one participant performed below chance, and although mean accuracy on the task was relatively high, nobody scored at ceiling.

### Literacy and grammaticality judgement

**Figure 4.**
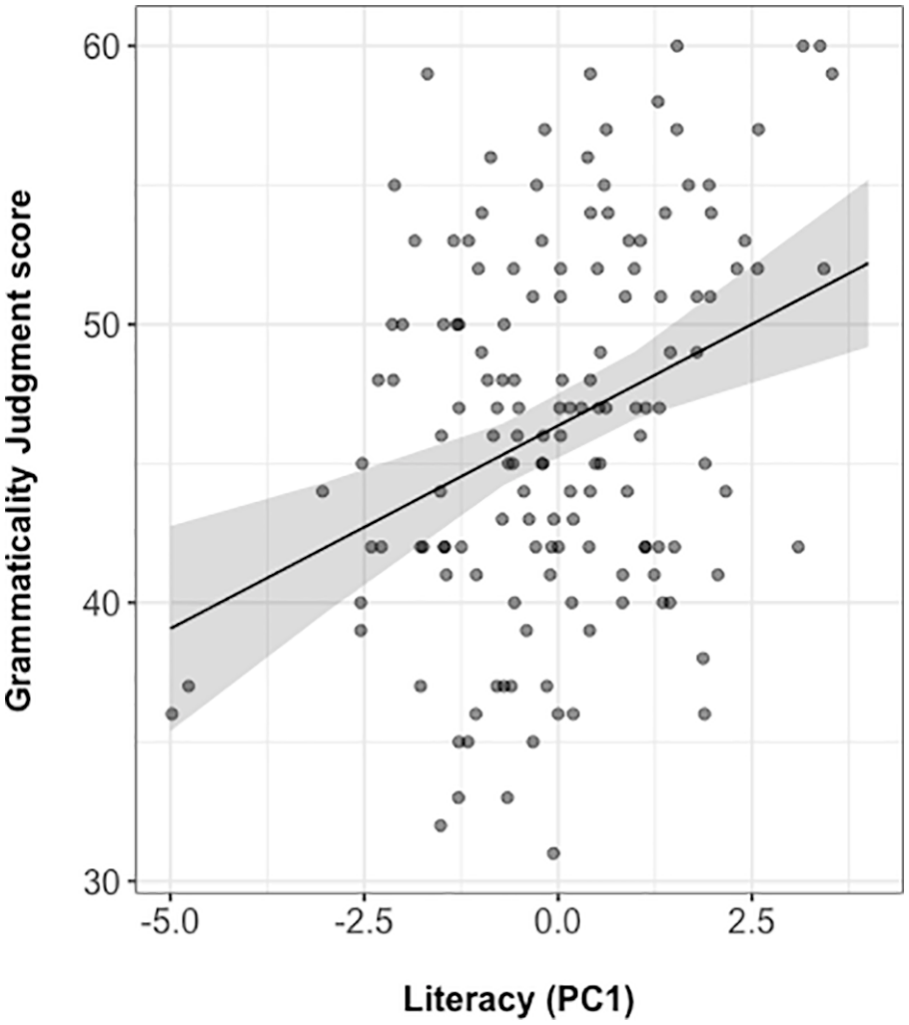
Scatterplot showing the relationship between literacy and grammaticality judgement. The line represents the regression fit from the model of judgement accuracy as a function of literacy score, controlling for the contribution of nonverbal IQ, verbal working memory, and processing speed (*R*^2^ = .211, effect size = .328). Dependent variable is raw score in the grammaticality judgement task (scored out of 64). PC1: first principal component; IQ: intelligence quotient.

We used multiple linear regression analysis to address our research question, “Are individual differences in literacy associated with the identification of grammatical norm violations in spoken language?” The approach (*lm* in R) enabled us to evaluate literacy as an independent predictor of grammaticality judgement while controlling for the contribution of nonverbal intelligence quotient (IQ), verbal working memory, and processing speed. Scores on the RPM, backward digit span, and letter comparison task were entered into the model as covariates, with literacy score as a predictor. The fitted model with an *R*^2^ of .211 revealed an independent contribution of literacy to participants’ grammaticality judgement accuracy (unstandardised β = 1.459, *SE* β = 0.358, 95% confidence interval = [0.756, 2.161], standardised β = 0.328). The standardised beta represents a measure of effect size, roughly equivalent to Pearson’s *r*. Figure 4 shows a scatterplot of the relationship between literacy and grammaticality judgement, and the line for the model is fitted to the data.

Regarding our secondary prediction that grammaticality judgements would be positively correlated with vocabulary knowledge, simple correlation analysis revealed a magnitude of *r* = .39 (Kendall’s τ = .25) between judgement accuracy and PPVT.

### Syntactic priming

Participants produced 2,499 PO responses (43.1%), 2,894 DO responses (49.9%), and 403 Other responses (6.9%). To evaluate the consistency of priming behaviour *within* individuals, we conducted a split-half reliability analysis. For subsets of even and odd trials separately, we calculated the proportion of PO responses as a function of prime condition for each participant. The PO proportions for the two subsets were then correlated to provide a measure of within-participant consistency. The spilt-half correlation magnitude was *r* = .66 (Kendall’s τ = .56), suggesting that priming behaviour was moderately consistent at the individual level.

**Figure 5.**
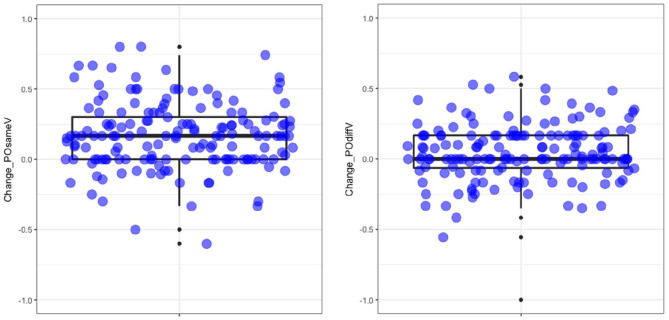
Box plot showing the distribution of difference scores for PO priming (i.e., proportion PO after PO primes, minus proportion PO at baseline). Left panel = same verb condition; right panel = different verb condition. Jittered data points correspond to individual participants’ difference scores. PO: prepositional-object.

There was considerable between-participant variability in priming behaviour across both structures and verb conditions. Figure 5 illustrates the individual variability in PO priming magnitude (and direction) for Same and Different verb conditions. The points below the zero line reflect a negative effect, that is, people who produced fewer POs in the priming conditions compared with baseline.

**Figure 6.**
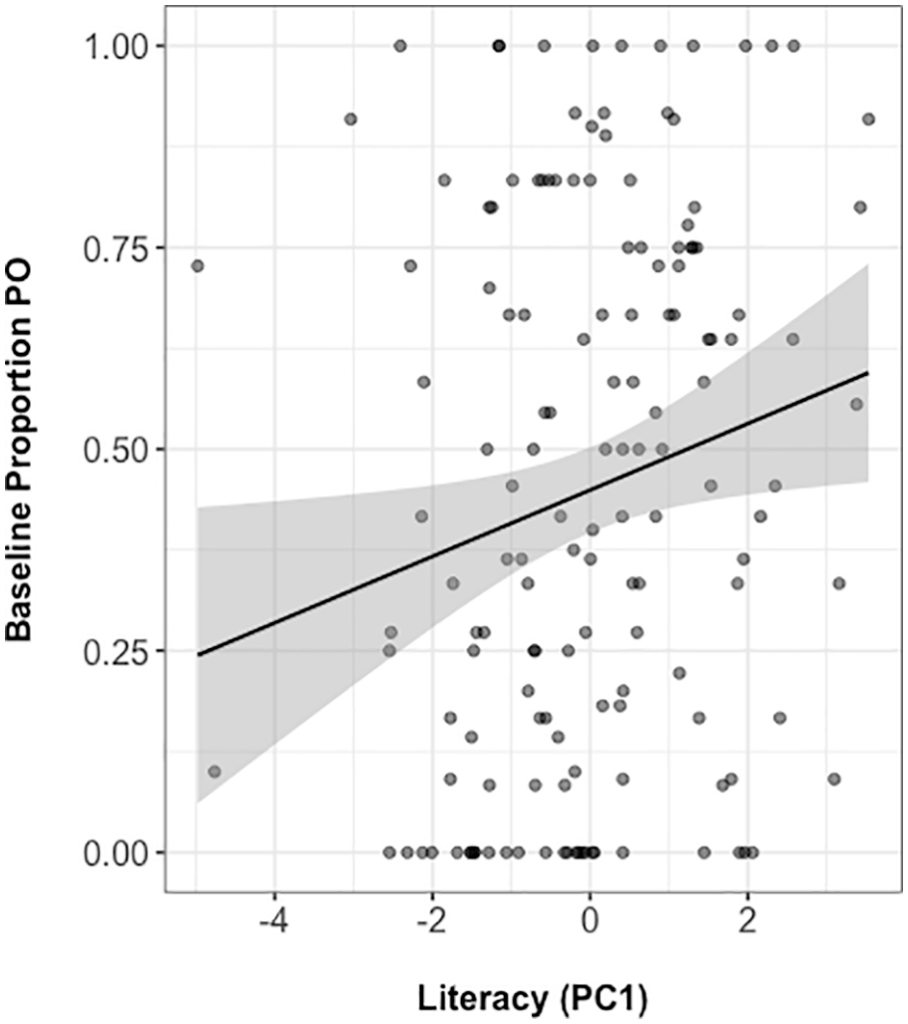
Literacy score plotted against proportion of POs produced in the baseline condition. PO: prepositional-object; PC1: first principal component.

We first explored whether literacy is associated with a bias towards PO or DO dative constructions in line with our prediction that written language experience affects the usage of structural alternates. Figure 6 shows that higher literacy scores are associated with producing a PO dative following a neutral baseline sentence (correlation coefficient, Kendall’s τ = .19). This is consistent with the notion that literacy experience affects (baseline) usage of PO or DO dative constructions.

Table 3 reports the proportion of POs and DOs out of all datives produced in each priming condition (excluding Other responses). The baseline proportions shown in Table 3 reflect the overall bias towards DO datives observed in this experiment (cf. Bernolet et al., 2016). The likelihood of producing a PO dative following a neutral baseline sentence was 45%. This increased to 51% when a PO prime was presented, resulting in a 6% priming effect in the absence of any lexical overlap between prime and target. When prime and target verbs were the same, there was a 62% chance of a PO response following a PO prime (17% priming effect). The 11% change in priming magnitude as a function of verb overlap demonstrates a lexical boost effect (see also the interaction between prime structure and verb condition shown in Table 4). DO datives showed weaker priming and lexical boost effects. Compared with baseline, the chance of a DO response was 4% higher in the different verb priming condition and 10% higher in the same verb condition, indicating a 6% lexical boost.

**Table 3.** PO and DO responses as a proportion of datives produced in the different priming conditions.

Prime condition	Proportion PO	Proportion DO
Baseline	0.45	0.55
PO Different Verb	0.51	0.49
PO Same Verb	0.62	0.38
DO Different Verb	0.41	0.59
DO Same Verb	0.35	0.65

PO: prepositional-object; DO: double-object.

**Table 4.** Summary of fixed effects in the mixed logit model (*N* = 4,984, log-likelihood = −2,425.7).

Predictor	Coefficient	*SE*	*z* value	CI
Intercept	−0.30	0.24	−1.25	[−0.78, 0.17]
Prime Type (Dative)	0.00	0.12	0.01	[−0.23, 0.24]
Prime Structure (PO)	1.27	0.10	12.68	[1.07, 1.46]
Verb Condition (Same)	0.18	0.09	1.92	[−0.00, 0.36]
Interaction = *Prime Structure & Verb Condition*	1.20	0.19	6.35	[0.83, 1.58]

The intercept represents the grand mean log-odds of a PO response, averaged across conditions. CI: confidence interval; PO: prepositional-object.

We fit a linear mixed effects logistic regression model to the participants’ responses across conditions, in line with the current standard for analysing categorical data (e.g., Barr et al., 2013; Jaeger, 2008). We used the “lme4” package in R version 1.0.153 (D. Bates et al., 2015; R Core Team, 2012) to create the model, which predicts the logit-transformed likelihood of a PO response (see Table 4). PO responses were as coded as “1” and DO responses were coded as “0” (other responses were excluded from this analysis). The first model comprised three fixed effects: Prime Type (Baseline/Dative), Prime Structure (PO/DO), and Verb Condition (Same/Different). We used contrast coding to capture the nested design, whereby structure and verb condition were manipulated only within the dative primes (not the baseline primes). In addition, we were interested in the interaction between Prime Structure and Verb Condition (i.e., the lexical boost effect). The model included random intercepts for participants and target verbs, as well as a random effect of Prime Structure by participant and by target verb, and a random effect of Verb Condition by participant. We assumed that the priming effect would be influenced to varying degrees by individual target verbs’ PO or DO bias, hence the inclusion of target verb in the model’s random effects structure. All random effects were de-correlated. The model results are summarised in Table 4.

Table 4 reveals a large syntactic priming effect (Prime Structure, *z* = 12.68). It also reveals a robust lexical boost effect (*Prime Structure & Verb Condition, z* = 6.35). These data therefore reflect a successful replication of the syntactic priming phenomenon (including the lexical boost effect) in a large, community-based sample of native Dutch speakers with varying literacy levels.

### Literacy and syntactic priming

**Figure 7.**
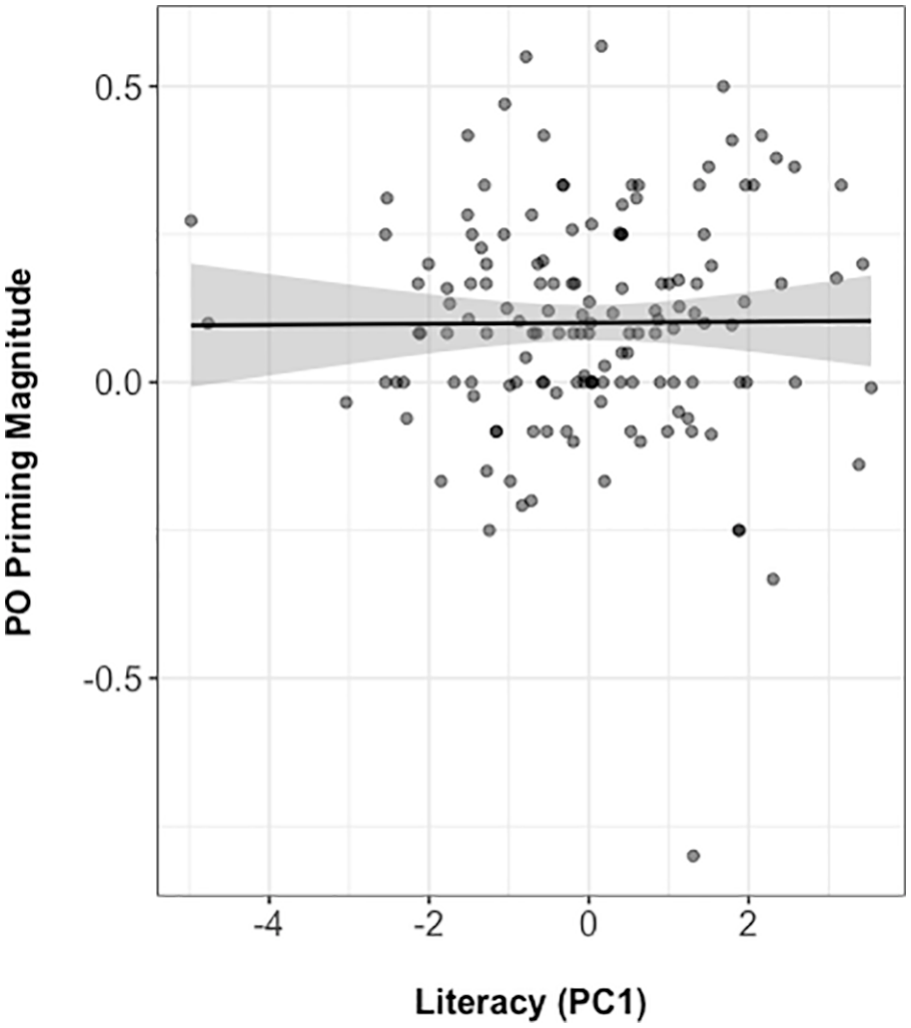
Literacy score plotted against priming magnitude (calculated as raw number of POs in PO prime condition minus POs at baseline). Correlation coefficient, Kendall’s τ = .081. PC1: first principal component; PO: prepositional-object.

Figure 7 plots literacy score against priming magnitude (calculated as raw number of POs in PO prime condition minus POs at baseline). It shows that literacy did not modulate syntactic priming.

We fit another mixed logit model to participants’ responses, this time incorporating Literacy (first principal component score) as a continuous predictor variable. The model results are summarised in Table 5.

**Table 5.** Summary of fixed effects in the mixed logit model (*N* = 4,984, log-likelihood = −2,422.8).

Predictor	Coefficient	*SE*	*z* value	CI
Intercept	−0.30	0.24	−1.27	[−0.77, 0.16]
Prime Type (Dative)	0.00	0.12	0.01	[−0.23, 0.24]
Prime Structure (PO)	1.26	0.10	12.67	[1.07, 1.46]
Verb Condition (Same)	0.18	0.09	1.95	[0.00, 0.36]
Literacy PC1	0.28	0.12	2.43	[0.05, 0.51]
Interaction = *Prime Structure & Verb Condition*	1.19	0.19	6.32	[0.82, 1.56]
Interaction = *Prime Structure & Literacy PC1*	0.00	0.07	−0.05	[−0.13, 0.12]

The intercept represents the grand mean log-odds of a PO response, averaged across conditions. CI: confidence interval; PO: prepositional-object; PC: principal component.

Table 5 reveals a large syntactic priming effect (Prime Structure, *z* = 12.67), which was not modulated by participants’ literacy skills (*Prime Structure & Literacy, z* = −0.05). Interestingly, the model shows that higher literacy scores are associated with a greater tendency to produce PO constructions in general (Literacy coefficient, *z* = 2.43, that is, a main effect of literacy on the log-odds of a PO response, averaged across conditions). This adds to the finding that literacy experience increases usage of PO dative constructions following a neutral baseline sentence (Figure 6). In other words, in our large, community-based sample (*N* = 161) of individuals with varying literacy levels, we observed that literacy experience affects the usage of Dutch PO or DO datives, yet literacy experience did not modulate the syntactic priming of these constructions.

Some accounts of syntactic priming predict an inverse effect of structural preference on priming. One way of examining this is via prime verbs’ individual subcategorisation biases (e.g., Bernolet & Hartsuiker, 2010; Jaeger & Snider, 2013). Another way is to look at the relationship between participants’ baseline structural preferences and their tendency to be primed. To the mixed logit model described in Table 4, we added PO base rate as a continuous predictor (PO base rate = rate of PO production as a proportion of dative responses in the baseline condition). Importantly, we included its interaction with PO prime to explore the question, “Does a lower PO base rate predict an increased likelihood of PO production after a PO prime?” The model results are summarised in Table 6. The confidence interval for the coefficient suggests only a very marginal interaction effect in the predicted direction, that is, people with a lower PO base rate show a tendency to prime more for PO.

**Table 6.** Summary of fixed effects in the mixed logit model (*N* = 4,984, log-likelihood = −2,286.9).

Predictor	Coefficient	*SE*	*z* value	CI
Intercept	−2.82	0.21	−13.74	[−3.22, −2.42]
Prime Type (Dative)	−0.02	0.12	−0.13	[−0.26, 0.22]
Prime Structure (PO)	1.01	0.23	4.35	[0.55, 1.46]
Verb Condition (Same)	0.16	0.10	1.54	[−0.04, 0.36]
PO base rate	5.45	0.22	24.38	[5.01, 5.89]
Interaction = *Prime Structure & Verb Condition*	1.21	0.19	6.50	[0.84, 1.58]
Interaction = *Prime Structure & PO base rate*	−0.67	0.37	−1.81	[−1.40, 0.05]

The intercept represents the grand mean log-odds of a PO response, averaged across conditions. CI: confidence interval; PO: prepositional-object.

Finally, to test our prediction that the strength of the lexical boost would be negatively associated with literacy, we created a further model, identical to the one described in Table 5, except for the addition of a three-way interaction between Prime Structure, Verb Condition, and Literacy PC1. We did not find evidence that literacy experience modulated the lexical boost, nor did the inclusion of the interaction improve model fit.

## General discussion

We investigated the contribution of individual differences in literacy experience to syntactic processes in spoken language. We administered a battery of tests to assess a range of literacy-related skills and their covariates (nonverbal IQ, verbal working memory, and processing speed). We used two experimental tasks, grammaticality judgement and syntactic priming, to target offline and online syntactic processes respectively. Four of our preregistered predictions were confirmed: Literacy was positively correlated with accuracy in an auditory grammaticality judgement task; vocabulary knowledge correlated positively with grammaticality judgement accuracy; participants produced more target completions containing the primed structure after hearing a prime sentence versus a structurally unrelated control sentence; and the likelihood of producing the primed structure was enhanced when the prime verb was repeated in the target sentence. Two of our preregistered predictions were not confirmed: There was no negative correlation between literacy and the magnitude of the syntactic priming effect observed, nor was there evidence for a stronger lexical boost in participants with lower literacy. We will now discuss these results in turn.

### Grammaticality judgement

Violations of four Dutch grammatical norms were tested. We observed systematic variation across individuals in their accuracy on the grammaticality judgement task. Above and beyond the contribution of nonverbal IQ, verbal working memory, and processing speed, literacy uniquely predicted participants’ ability to correctly accept and reject spoken sentences according to the prescriptive grammatical norms of their language. Controlling for the contribution of nonverbal IQ, verbal working memory, and processing speed, we observed a robust effect (standardised β = 0.33) of literacy experience on the detection of grammatical norm violations in spoken sentences, suggesting that exposure to the syntactic complexity and diversity of written language has specific benefits for general (modality-independent) syntactic knowledge. This result converges with and extends previous findings concerning the relationship between print exposure and syntactic abilities (Dąbrowska, 2012; Street & Dąbrowska, 2010). The current study used a multifaceted measure of literacy experience and found a moderate positive correlation with metalinguistic syntactic abilities, adding evidence to support the link between language experience and aptitude in adult native speakers. Our separate finding that grammaticality judgement was positively correlated with vocabulary knowledge in adult native speakers is consistent with longitudinal evidence for the intertwined development of grammar and vocabulary (E. Bates et al., 1995; Hayiou-Thomas et al., 2006).

In line with the notion that greater vocabulary knowledge is associated with greater written language experience, we had preregistered that vocabulary knowledge will be positively correlated with accuracy in grammaticality judgements. We had also preregistered to investigate the influence of verbal working memory as a covariate (https://osf.io/zykp2). One may however regard verbal working memory more accurately as a secondary influence of reading (Huettig & Pickering, 2019, cf. Demoulin & Kolinsky, 2016; Smalle et al., 2019). Secondary influences can also be attained by listening to “book-like” auditory materials. Listening to audiobooks, for example, supports the acquisition of “book language” because it contains syntactically more elaborate language (with higher demands on verbal memory) and more extensive and sophisticated vocabulary than conversational speech. Primary influences are those that are more directly linked to the physical act of reading (e.g., efficient decoding of written language; increased exposure to the extreme form-invariance of printed word forms; parallel processing of multiple letters/words in proficient readers; see Huettig & Pickering, 2019, for further discussion). The literacy effect on grammaticality judgement in our study may well be more secondary in nature, likely originating from exposure to “book language” as opposed to physical reading practice. Future research thus could usefully delineate primary and secondary influences of literacy directly by conducting a confirmatory preregistered study.

From an experience-based perspective, we had a straightforward prediction about the effect of literacy experience on grammaticality judgement accuracy. The task was to judge the “correctness” of spoken sentences with reference to prescriptive norms that are attested in written texts far more consistently than in spoken language. Therefore, on the basis of quantitative and qualitative differences in the input, prolific readers should have more relevant data to support their judgements.

When considered as a measure of explicit syntactic awareness, grammaticality judgement requires the caveat that the contribution of some implicit syntactic knowledge to task performance cannot be ruled out. Given that no psycholinguistic task is “purely” explicit or implicit, and that participants in grammaticality judgements are explicitly asked to make a metalinguistic judgement, we can however be reasonably confident that grammaticality judgements involve more explicit processing than the syntactic priming task that only requires participants to complete sentences (and does not explicitly draw attention to the purpose of the task).

### Syntactic priming

We successfully replicated the most well-documented effects in the syntactic priming literature. Using comprehension-to-production priming of the dative alternation in Dutch, we observed a statistically large syntactic priming effect (*z* = 12.67) and a robust lexical boost effect (*z* = 6.35) in a large, community-based sample of native Dutch speakers with varying literacy levels.

Within our sample (*N* = 161), there was considerable individual variability in syntactic priming behaviour, with many participants showing no priming at all, and others showing a negative effect. Individual differences have been given little attention in the syntactic priming literature to date (cf. Kidd, 2012). There is a tendency to consider effects only at the group level and to dismiss the absence of priming as experimental noise. Gathered from a large and diverse sample (with respect to ability), our data suggest that between- and, to some extent, within-participant variability is the norm rather than the exception for syntactic priming.

The large syntactic priming effect we observed was not modulated by participants’ literacy experience (*Prime Structure & Literacy, z* = −0.05). Importantly, this absence of a modulation of the priming effect was not due to an absence of differences in structure *usage*. Our model revealed that higher literacy scores were in fact associated with a greater tendency to produce PO constructions in general (Literacy coefficient, *z* = 2.43, that is, a main effect of literacy on the log-odds of a PO response, averaged across conditions). Moreover, literacy experience was associated with increased usage of PO dative constructions (and therefore decreased usage of DO datives) following a neutral baseline sentence (Figure 6). We therefore conclude that literacy experience affects the usage of Dutch PO/DO dative alternates but does not modulate the syntactic priming of these constructions.

We had predicted a negative correlation between literacy experience and priming magnitude, motivated by the notion that literacy-related differences in usage of the dative alternation would affect syntactic priming of the structures (cf. Huettig et al., under review). This hypothesis was not supported. One possibility is that literacy-related usage differences only play a role in syntactic priming during the stage of language *acquisition* but (more or less) “level off” in proficient language users such as the adults who took part in the present study. Future (large *N*) studies could usefully further explore this possibility. Given that our participants may have reached a plateau in L1 syntactic acquisition, regardless of literacy level, a large-scale developmental study may be particularly fruitful for exploring the influences of literacy on syntactic priming in childhood.

Our preregistered prediction that participants with less literacy experience would show a stronger lexical boost effect was also not borne out in the data. Despite the robust lexical boost observed at the group level, there was no indication that it was modulated by individual differences in literacy experience. Based on the account presented by Huettig et al. (under review), inexperienced literates might be expected to show a larger lexical boost because items (in this case, verbs) that are encountered less often (e.g., due to limited written input) are argued to have a lower resting state and thus receive a larger boost from the same amount of activation. One possible reason why we did not observe such a relationship in the current study is that relatively high frequency verbs were used. If the resting state of the prime verbs was generally high due to frequent exposure, it follows that the activation boost caused by a given verb’s repetition (i.e., the lexical boost) would not be sensitive to the effects of differential written language input. Future studies could explore this hypothesis by testing low-frequency verbs, which would be expected to have a relatively low resting state in people who read very little.

In our experiment, PO datives were produced less frequently than DO datives overall, yet yielded a larger priming effect. Previous studies have shown that infrequent structures tend to prime more reliably, in line with notions that the unexpectedness of the prime structure has a significant effect on priming (i.e., more frequent/predictable structures are assumed to prime less than relatively infrequent/unpredictable structures, the so-called inverse preference effect). Our measure of participants’ PO/DO preference following a nondative prime (baseline condition) allowed us to test this notion. Our exploratory analysis revealed a marginal negative effect of PO base rate on PO priming, suggesting a small tendency in the predicted direction. However, given the big sample size, our data do not provide robust evidence for the inverse preference effect, as has been reported elsewhere in the literature (e.g., Jaeger & Snider, 2013). Given that many other (often unpublished) studies have failed to observe the inverse preference effect (e.g., Kaschak & Borreggine, 2008), future research could be directed at exploring which factors modulate the presence or absence of this effect in priming experiments.

We can reject some alternative explanations of the present data with considerable confidence. Is the absence of a modulation of priming magnitude by literacy “just a null effect” in one study that failed to detect a “real” effect? Given the large (preregistered) sample size, heterogeneity of participants in terms of literacy level, and carefully selected literacy measures, such an alternative interpretation is very unlikely to be correct. Another suggestion might be that we should have relied on corpus measures to estimate the distribution of PO/DO datives in spoken and written Dutch corpora. We conjecture that measuring PO/DO usage directly with a baseline as in the present study is the more reliable way of assessing influences of written language experience. Note that Dutch corpora (Colleman, 2009; Haemers, 2012) and experimental baseline measures (e.g., Bernolet et al., 2016) are often inconsistent with respect to PO/DO distributions (Hartsuiker, personal communication, June 2020). This is likely because both Dutch corpora and experiment samples tend to be small in size and prone to bias. It is noteworthy, for instance, that the overall distribution of PO and DO datives in our data was quite different from previous Dutch priming studies (e.g., Bernolet et al., 2016). We found DO to be marginally the more frequent construction, in contrast to the strong PO bias previously reported by Bernolet and colleagues. This divergence likely reflects differences in sample size but also in the populations sampled: In our study, highly experienced literates, like Bernolet’s undergraduate participants, did demonstrate an overall PO bias, whereas less experienced literates tended to produce more DO datives. Moreover, unlike the “averages” in corpus studies, we found that for the *same* people, literacy experience modulated usage (in line with Montag & MacDonald, 2015, who observed that the spoken production of experienced readers echoes the structural distributions of written language), but not priming of the PO/DO dative alternation in Dutch.

Finally, in the present study, two different measures were used to tap into explicit and implicit syntactic processes. We acknowledge that grammaticality judgement is more of an offline task than syntactic priming (although both have implicit and explicit components). Thus, the two types of processes may have been indexed by their respective measures to different extents. One could argue that the observed contribution of literacy experience to explicit but not implicit syntactic processes was simply an artefact of the different measures used. Note, however, that this is not what we conclude here. Our conclusion is more specific, namely, that long-term written language experience affects syntactic awareness (as indexed by grammaticality judgement) and usage but not the syntactic priming of spoken sentences (as measured by the syntactic priming paradigm). It is noteworthy that we (Favier et al., under review) have recently found that literacy affects (largely) implicit syntactic prediction in spoken sentence processing. It will be interesting for further research to explore why (largely) implicit syntactic prediction but not (largely) implicit syntactic priming is affected in spoken sentence processing.

## Conclusion

We conducted a large-scale correlational study with 161 adult native speakers of Dutch to examine literacy experience as a predictor of syntactic processing in spoken language while controlling for the contribution of nonverbal IQ, verbal working memory, and processing speed. As predicted, we found an effect of literacy on explicit syntactic awareness, specifically the detection of grammatical norm violations in spoken sentences. Literacy was also related to the usage of Dutch (PO vs. DO) datives but, contrary to our prediction, had no detectable effect on their implicit syntactic priming in our adult sample. Further research is needed to investigate why usage but not priming of syntactic structures is modulated by lifelong syntactic experience in proficient language users.

## Notes

1. Stimuli for both experimental tasks, as well as the data, and other further analyses are available in the supplementary materials on https://osf.io/eg7pw/?view_only=56ebe093366943bfb3e6f3eb995c4fac

2. To interpret our results, we adopt a magnitude estimation approach where applicable, in line with recommendations that the field should be shifting focus away from significance testing, towards estimation based on effect sizes and confidence intervals instead (Cumming, 2013; see also Hintz et al., 2017; Huettig & Janse, 2016).
